# Overlap in meaning is a stronger predictor of semantic activation in GPT-3 than in humans

**DOI:** 10.1038/s41598-023-32248-6

**Published:** 2023-03-28

**Authors:** Jan Digutsch, Michal Kosinski

**Affiliations:** 1 Leibniz Research Centre for Working Environment and Human Factors at the Technical University of Dortmund, Dortmund, Germany; 2 Stanford University, Stanford, CA 94305 USA; 3 Institute of Behavioral Science and Technology, University of St. Gallen, St. Gallen, Switzerland

**Keywords:** Human behaviour, Psychology

## Abstract

Modern large language models generate texts that are virtually indistinguishable from those written by humans and achieve near-human performance in comprehension and reasoning tests. Yet, their complexity makes it difficult to explain and predict their functioning. We examined a state-of-the-art language model (GPT-3) using lexical decision tasks widely used to study the structure of semantic memory in humans. The results of four analyses showed that GPT-3’s patterns of semantic activation are broadly similar to those observed in humans, showing significantly higher semantic activation in related (e.g., “lime–lemon”) word pairs than in other-related (e.g., “sour–lemon”) or unrelated (e.g., “tourist–lemon”) word pairs. However, there are also significant differences between GPT-3 and humans. GPT-3’s semantic activation is better predicted by similarity in words’ meaning (i.e., semantic similarity) rather than their co-occurrence in the language (i.e., associative similarity). This suggests that GPT-3’s semantic network is organized around word meaning rather than their co-occurrence in text.

## Introduction

Modern large language models (LLMs) employ artificial neural networks that generate texts virtually indistinguishable from those written by humans and achieve near-human performance in comprehension and reasoning tests (e.g.,^1,2^). LLMs are not provided with grammar rules or dictionaries, but are repeatedly presented with a fragment of text (e.g., a Wikipedia article) with one word removed (e.g., “Paris is the capital of _____”), and have to predict the missing word. In the training process, typically involving trillions of trials drawn from huge text corpora, LLMs become skilled language users, spontaneously discovering linguistic rules and word associations.

LLMs’ complexity means that it is difficult to explain their functioning and anticipate their future behavior. Both users and creators are often surprised by their emergent properties, both useful (e.g., the ability to translate between languages or write computer code;^3^) and problematic (e.g., gender and racial biases;^1^). It is also unclear whether they are mere stochastic parrots^4^ limited to modeling word similarity^5^, or if they recognize concepts and could be ascribed with some form of understanding of the meaning of the words they so skillfully use.

The challenge of understanding complex LLMs is not new. The last decades brought significant progress in understanding a much more complex entity capable of generating and comprehending language: the human brain. The methods used to better understand the human brain can be adapted for studying the artificial brain, and there is a growing interest in doing so (e.g.,^6,7^).

To understand and produce language, humans utilize semantic memory that stores information about words and their meaning^8–11^. To unravel the structure of semantic memory, researchers often study the patterns of *semantic activation*, or the phenomenon in which exposure to one word facilitates the processing and retrieval of other words^12^. For example, when asked “What do cows drink?,” people tend to answer “milk” instead of “water” (only calves drink milk), revealing that “cow” and “drink” activate “milk” in semantic memory. The research reveals that semantic activation occurs mostly between words that often co-occur in the language (i.e., associative similarity; “wrong–way”) and words with overlapping meaning (i.e., semantic similarity; “annoying–irritating”;^13,14^). Moreover, while semantic and associative similarity often goes hand in hand, activation readily spreads between the words of similar meaning that rarely co-occur in the language (i.e., purely semantically related words, such as “cow” and “sheep”). This suggests that purely semantically related words are closely connected in the semantic memory, likely through their mutual connections to simple concepts. “Cow” and “sheep,” for example, are both linked with “horns,” “milk,” and “farm”^10,15^.

The present research aims to contribute to our understanding of the structure of the semantic memory of humans and LLMs by comparing their patterns of semantic activation. In humans, semantic activation is typically measured using *semantic priming*, where the exposure to one word (i.e., *prime*) facilitates the processing and retrieval of another word (i.e., *target*). Semantic priming is commonly measured using *lexical decision tasks*^8^, where participants are presented with a prime (e.g., “lemon”) followed by a real word (e.g., “lime”) or a non-word (e.g., “leton”). Participants have to decide, as quickly and accurately as possible, whether the second word is a real word. Their speed and accuracy are interpreted as a proxy for semantic activation. For example, when preceded by “lemon,” “lime” is more quickly recognized as a real word than when it is preceded by “dog”.

In LLMs, semantic activation can be measured directly from words’ distances in the model’s lexical space. Early models derived lexical space from word co-occurrences in the training data (e.g.,^16^). They were followed by models capturing words’ context in the training data (e.g.,^17,18^). Most recent LLMs employ dynamic lexical space that changes depending on the word’s context in a given task (e.g.,^19^). Studies comparing language models’ and humans’ lexical spaces show that they are increasingly similar as the models become more complex (e.g.,^20–22^).

The present research compares semantic activation patterns between humans and OpenAI’s Generative Pre-trained Transformer 3 (GPT-3;^1^), using word pairs typically used in human studies^23^. Analysis 1 compares the semantic activation of GPT-3 and humans across three semantic relatedness conditions and shows that GPT-3’s semantic activation patterns broadly mirror those observed in humans. Analyses 2 and 3 compare semantic activation across 12 types of prime-target associations and show that, when compared with humans, GPT-3’s lexical space is more strongly organized around semantic (rather than associative) similarity. Finally, analysis 4 compares the relative importance of semantic versus associative activation across five GPT-3 variants as well as three other language models. It shows that the newer the model, the more its semantic space is organized around the semantic similarity.

## Methods

Lexical decision tasks (n = 6646) and human participants’ responses (n = 768 students from three universities) were obtained from the Semantic Priming Project database (^23^; https://www.montana.edu/attmemlab/spp.html). As it is publicly available and de-identified, its analysis does not constitute human subject research.

Lexical decision tasks consist of a target word (e.g., “lemon”) matched with three primes (first-associate, other-associate, and unrelated). Human participants’ response times were standardized for each participant separately and then averaged for each word pair. Following Mandera et al.^24^, we excluded all non-word trials, erroneous responses, and trials with reaction times deviating more than three standard deviations from the within-person mean.

We used GPT-3’s most recent engine aimed at capturing words’ similarity (“text-embedding-ada-002”; all settings were left at their default values). It employs a 1536-dimensional semantic space. The location of words or phrases in this space is described by 1536-value-long numerical vectors (i.e., embeddings). In analysis 4, we additionally used four older GPT-3 variants ("text-similarity-[ada/babbage/curie/davinci]-001"), as well as older language models ("xlm-roberta-base", "albert-base-v2";^25,26^).

Semantic activation in GPT-3 was operationalized as the cosine distance between prime and target words’ embeddings. Cosine similarity is similar to the correlation coefficient, ranging from − 1 (dissimilar) to 1 (similar). The cosine similarity between “lime” and “lemon,” for example, equals 0.35, which is much closer than the similarity between “tourist” and “lemon” (− 0.03). Note that this measure of activation is non-directional: “lime” activates “lemon” as much as “lemon” activates “lime”.

We considered an alternative approach, previously used by Misra et al.^19^: presenting GPT-3 with prime words (or sentences containing the prime words) and recording the probability distribution of possible completions. Yet, we believe that this strays too far from the original format of the lexical decision task, which is context-free and does not require participants to complete word sequences. Moreover, the context surrounding the prime becomes a confound, even if it is a mere punctuation sign. The probability of “race” significantly differs among “car race” (log(P) = − 10.27), “car, race” (log(P) = − 9.29), and “car-race” (log(P) = − 6.52).

Separately for humans and GPT-3, semantic activation scores across all prime-target pairs (i.e., first-associate, other-associate, and unrelated) were standardized (mean of 0 and standard deviation of 1) before conducting statistical analysis. To facilitate visual comparisons between humans and GPT-3, the semantic activation displayed was converted to percentiles on the plots.

## Results

### Analysis 1

We first compare the semantic activation between humans and GPT-3 across three prime word types: The *first-associate prime* (e.g., “lime”) is a word to which the target is the most common response (e.g., “Which word comes first into your mind when hearing lemon?”); an *other-associate prime* (e.g., “sour”) is a randomly selected word to which the target is a less common response; and an *unrelated prime* (e.g., “tourist”) is a randomly selected word to which the target has not appeared as a response (and vice versa).

**Figure 1:**
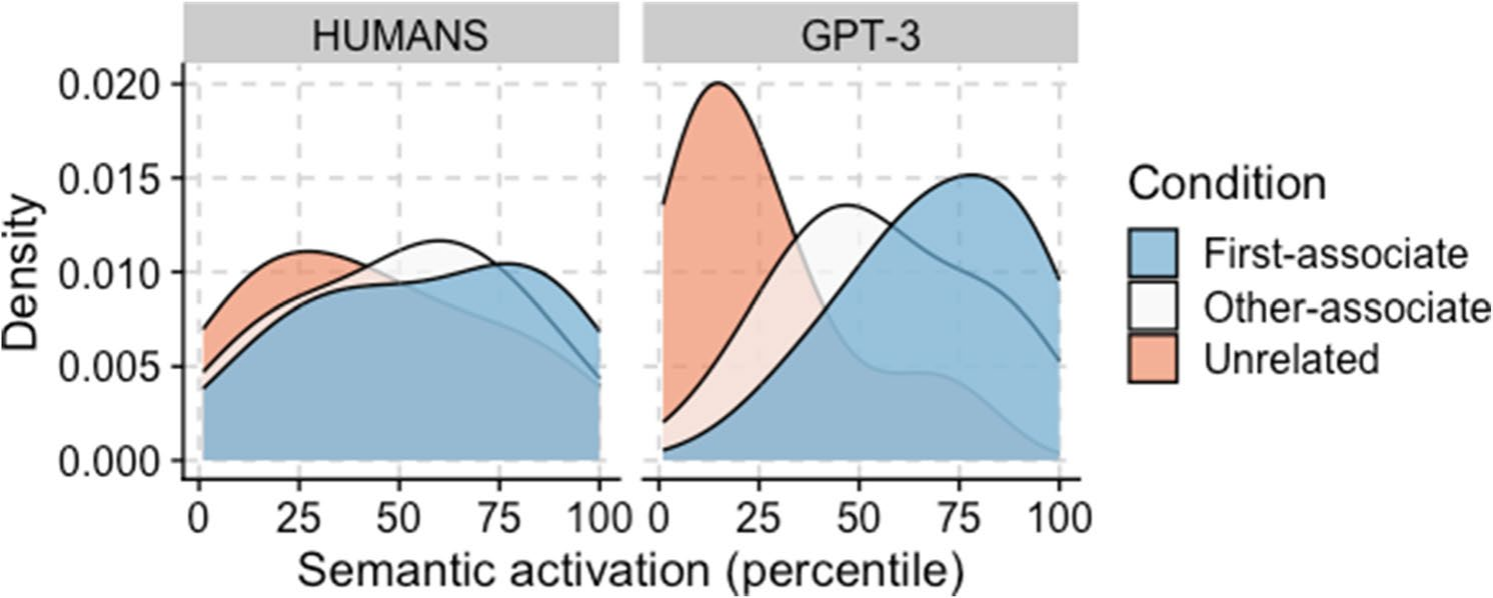
Semantic activation across priming conditions.

Figure 1 presents the density distribution of semantic activation for humans (left panel; approximated by the lexical decision task reaction times) and for GPT-3 (right panel; approximated by the cosine similarity between prime and target word embeddings). Like human respondents, GPT-3 shows the highest semantic for first-associate word pairs, followed by other-associates and unrelated word pairs.

However, the differences in mean semantic activation between the priming conditions were larger for GPT-3 than for humans (ANOVA’s F(26511) = 1343.70, *p* < 0.001; η^2^ = 29.22% or a large effect size versus F(26511) = 104.76, *p* < 0.001; η^2^ = 3.12% or a small effect size;^27^). This is to be expected, as GPT-3’s semantic activation is measured directly (via cosine similarity), while in humans it is approximated (via semantic priming). Moreover, in contrast with humans, GPT-3 does not suffer from inattention, fatigue, and other response biases. Therefore, GPT-3’s results are expected to be more pronounced across all our analyses.

### Analysis 2

The results of Analysis 1 showed that semantic activation patterns in GPT-3 broadly mirror those observed in humans, but the effect of the priming condition is stronger for GPT-3. Here, we take a closer look by comparing semantic activation across 12 types of prime-target word pairs, following the classification used by the Semantic Priming Project database^23^.

**Figure 2:**
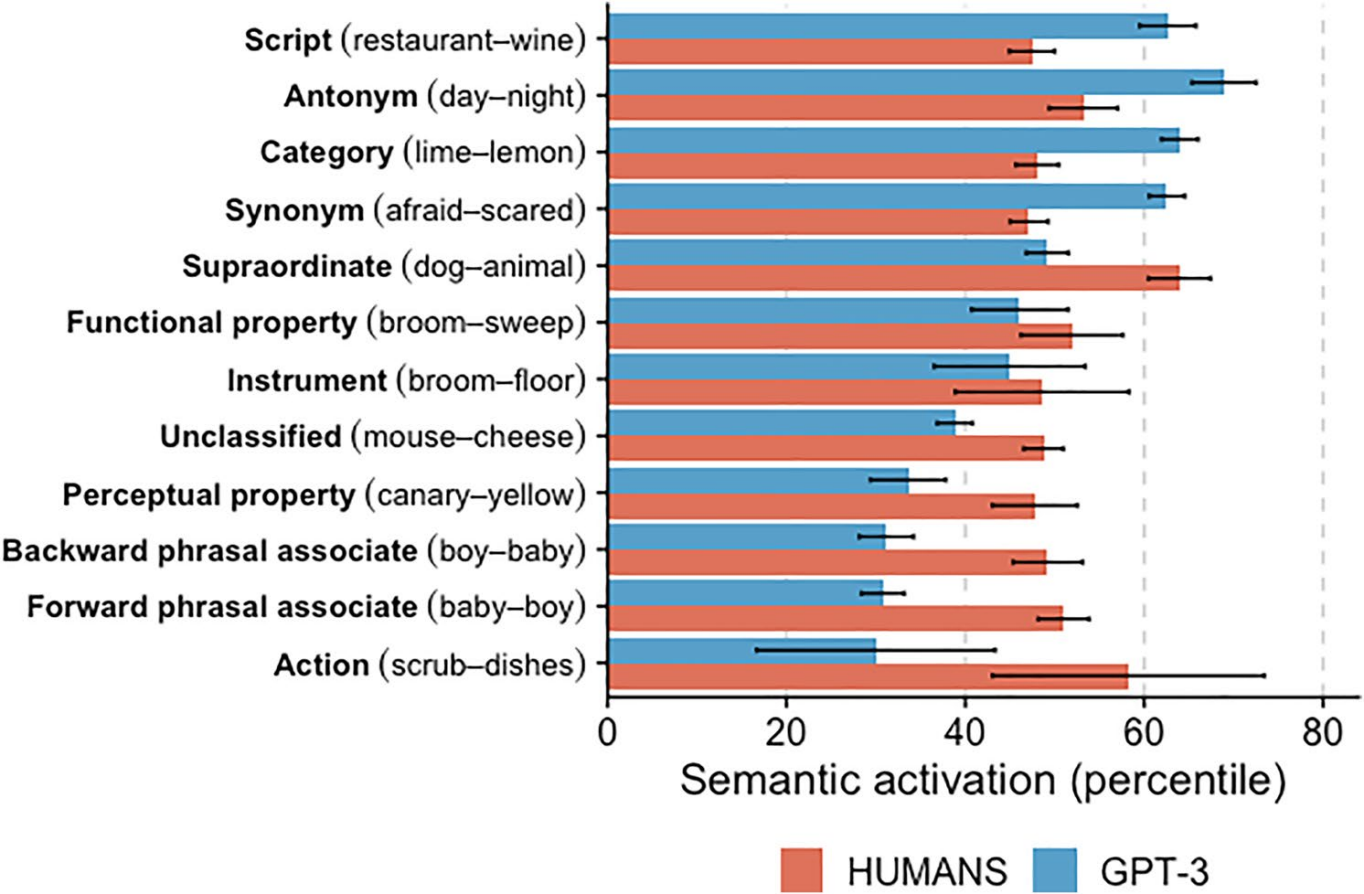
Semantic activation and prime-target association type. Word pairs in brackets are examples. Error bars represent 95% confidence intervals.

The results presented in Fig. 2 show clear differences between semantic activation in humans (red bars) and GPT-3 (blue bars). While for humans semantic activation did not depend strongly on semantic association, clear differences were observed for GPT-3. Its semantic activation was strongest for script (68th), antonyms (68th), categories (63rd), and synonyms (62nd) and weakest for backward and forward phrasal associates (30th and 29th, respectively) and action (28th).

This indicates that in GPT-3 (but not in humans), semantic activation was strongly driven by semantic rather than associative similarity. Antonym, synonym, and category word pairs share many semantic features (e.g., lime and lemon are both sour fruits of similar shape;^14,15^) and relatively rarely co-occur in language. In contrast, forward and backward phrasal associates share little semantic similarity but often co-occur in language.

### Analysis 3

Analysis 2 showed an interesting difference between humans and GPT-3: GPT-3’s lexical space seems to be organized more strongly around semantic similarity than in humans. Yet, as in Analysis 1, the clearer pattern of GPT-3’s results could be driven by the direct approach to measuring its semantic activation. We further explore this issue by comparing semantic activation between GPT-3 and humans on the level of individual word pairs.

Table 1 presents word pairs with the largest differences in semantic activation between GPT-3 and humans. For example, “introvert” highly activates “extrovert” in GPT-3 (3.81 SD above the mean), but not in humans (3.06 SD below the mean).

**Table 1:** Largest differences in semantic activation between humans and GPT-3.

Prime	Target	Association type	Z-score	
			Humans	GPT-3
Introvert	Extrovert	Antonym	− 3.06	3.81
Understand	Misunderstand	*Antonym*	− 5.23	0.89
Clumsy	Klutz	Synonym	− 5.65	0.45
Britannica	Britain	Unclassified	− 3.57	2.40
England	Britain	Synonym	− 2.94	2.99
Quality	Characteristic	*Synonym*	− 6.63	− 0.70
Outgoing	Extrovert	Synonym	− 4.28	1.42
Advise	Advice	Unclassified	− 1.64	4.00
Pro	Con	Antonym	− 3.71	1.73
Trait	Characteristic	*Synonym*	− 5.52	− 0.12
Crutch	Leg	Instrument	1.82	− 1.21
Ribs	Broken	Unclassified	1.76	− 1.10
Mountain	Top	Forward phrasal associate	1.84	− 0.98
Brake	Go	Antonym	1.40	− 1.32
Braces	Young	Unclassified	1.22	− 1.48
Approval	Yes	Unclassified	2.00	− 0.64
Clerk	Person	Unclassified	1.69	− 0.92
Real	People	Forward phrasal associate	1.35	− 1.26
Acre	Land	Supraordinate	1.56	− 1.04
Loud	Pain	Unclassified	1.21	− 1.40

Association types printed in regular font come from the Semantic Priming Project database; those printed in *italics* were missing and were added by us.

The results confirm the results of Analysis 2. Compared with humans, GPT-3 is particularly prone to be activated by semantically similar word pairs such as antonyms (e.g., “introvert–extrovert” and “understand–misunderstand”) and synonyms (e.g., “clumsy–klutz” and “england–britain”). Humans, on the other hand, are relatively more driven by associatively similar words, such as forward phrasal associates (e.g., “mountain–top” and “real–people”).

### Analysis 4

Analyses 1–3 indicated that, when compared with humans, the semantic space of the most recent engine of GPT-3 was organized around semantic, rather than associative relatedness. Here we compare the relative importance of the associative versus semantic relatedness across several LLMs. Associative relatedness was approximated by averaging the semantic activation for forward and backward phrasal associates. Semantic relatedness was estimated by averaging the semantic activation for antonyms, synonyms, and categories (see "Analysis 2" Section and Fig. 2).

**Figure 3:**
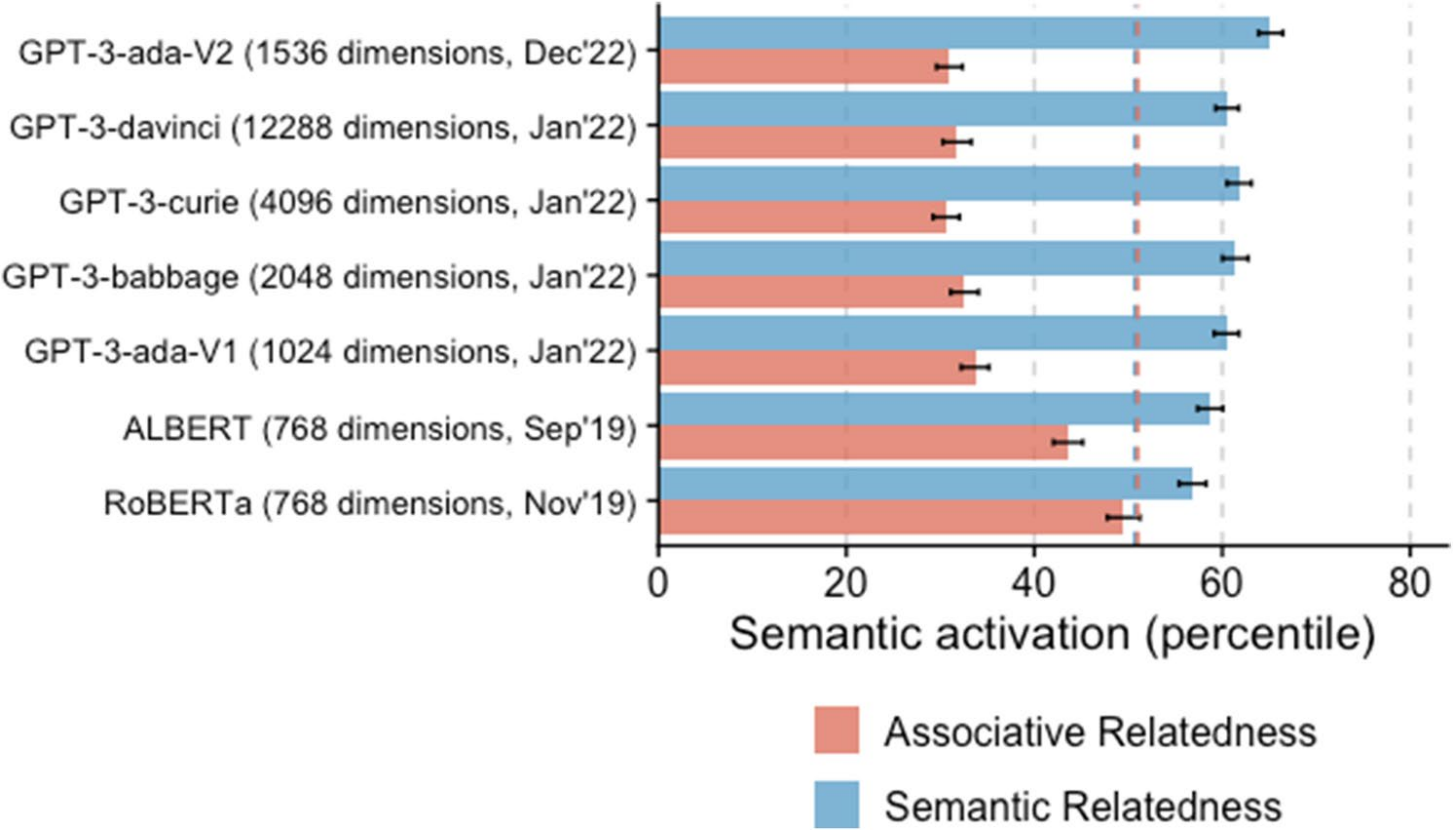
Semantic activation for associatively (red) and semantically (blue) related words. Dotted lines represent semantic activation for the associatively (red) and semantically (blue) related words in humans.

The results, presented in Fig. 3, show that the more recent and more complex the model the more its semantic space is organized around semantic rather than associative relatedness.

## Discussion

Our results show that semantic activation in GPT-3 broadly mirrors those observed in humans. GPT-3’s semantic activation was significantly higher for first-associated (e.g., “lime–lemon”) word pairs than other-associated (e.g., “sour–lemon”) or unrelated (e.g., “tourist–lemon”) word pairs (Analysis 1). However, the analysis of prime-target association types in Analysis 2 revealed that GPT-3’s semantic activation is more strongly driven by the similarity in words’ meaning (i.e., semantic similarity) than their co-occurrence (i.e., associative similarity). This effect is stronger in GPT-3 than in humans. In fact, in Analysis 3, the most drastic differences in semantic activation between GPT-3 and humans were observed for synonyms (more similar according to GPT-3) and phrasal associates (more similar according to humans). This suggests that semantic similarity is a stronger predictor of semantic activation in GPT-3 than in humans. Moreover, Analysis 4 reveals that the role of semantic similarity in predicting semantic activation is greater in the more complex and more recent models.

That semantic activation occurs both in humans and GPT-3 is unsurprising. As humans are affected by their semantic activation patterns while generating language, models trained to do the same would benefit from possessing—or simulating—a similar mechanism. It is also possible that the spreading activation (see^28^) is an inherent property of any complex neural network aimed at generating human-like language. To some extent, LLMs may be mirroring (at least on the functional level) semantic structures present in humans. GPT-3’s susceptibility to semantic activation is not the first example of neural networks mirroring human-like psychological processes. Past research has shown, for example, that LLMs mirror human gender and ethnic biases^1^, and neural networks trained to process images suffer from human-like optical illusions^29,30^. None of those functions were engineered or anticipated by their developers.

What is surprising, however, is the relatively larger importance of semantic similarity for GPT-3 and the relatively larger importance of associative similarity for humans. It is possible that it is an artifact of the measurement approach used in humans and GPT-3. Semantic priming effects measured using lexical decision tasks in humans are likely affected by processes beyond semantic activation, such as *expectancy* (i.e., an intentional generation of potential completions of a word sequence;^31^) or *semantic matching* (i.e., a retrospective search for the target-prime relationship;^32^). This could be a potential source of noise or bias that is not present in the more direct measure of semantic activation applied to GPT-3 (cosine distance).

Studying LLMs such as GPT-3 could boost our understanding of human language. LLMs are trained to mimic human behavior and could be used as model participants in psycholinguistic studies, enabling researchers to quickly and inexpensively test hypotheses that could be later confirmed in humans (see^33^ for a recent example). Unlike humans, LLMs do not suffer from fatigue and lack of motivation, and can respond to thousands of tasks per minute. Moreover, artificial and biological neural networks aimed at processing language may have convergently evolved similar neural structures. As artificial neural structures are easier to study than biological ones, studying LLMs could further the understanding of mechanisms and processes occurring in the human brain. This is not a new idea: The structures of the artificial neural networks trained to process images mirror those observed in the ventral visual pathway^34^. More broadly, the study of convergent evolution has greatly benefited biology, neuroscience, psychology, and many other disciplines (e.g.,^35^). Yet, we should tread carefully: As our results illustrate, LLMs’ behaviors are sometimes significantly different from those observed in humans, despite their superficial similarities.

## Data Availability

Data and code used in the analyses can be found at https://psyarxiv.com/dx5hc.
